# Gene-specific mitochondria dysfunctions in human *TARDBP* and *C9ORF72* fibroblasts

**DOI:** 10.1186/s40478-016-0316-5

**Published:** 2016-05-05

**Authors:** Elisa Onesto, Claudia Colombrita, Valentina Gumina, Maria Orietta Borghi, Sabrina Dusi, Alberto Doretti, Gigliola Fagiolari, Federica Invernizzi, Maurizio Moggio, Valeria Tiranti, Vincenzo Silani, Antonia Ratti

**Affiliations:** Department of Neurology and Laboratory of Neuroscience, IRCCS Istituto Auxologico Italiano, Via Zucchi, 18, Cusano Milanino, 20095 MI Italy; Department of Pathophysiology and Transplantation, ‘Dino Ferrari’ Center - Università degli Studi di Milano, Milan, Italy; Laboratory of Immunorheumatology, IRCCS Istituto Auxologico Italiano, Milan, Italy; Department of Clinical Sciences and Community Health, Università degli Studi di Milano, Milan, Italy; Molecular Neurogenetics Unit, Foundation IRCCS-Neurological Institute “Carlo Besta”, Milan, Italy; Neuromuscular and Rare Disease Unit, Department of Neuroscience, Foundation IRCCS Ca’ Granda Ospedale Maggiore Policlinico, Università degli Studi di Milano, Milan, Italy

**Keywords:** ALS, FTD, TDP-43, C9ORF72, Fibroblast, Mitochondria dysfunction

## Abstract

Dysregulation of RNA metabolism represents an important pathogenetic mechanism in both amyotrophic lateral sclerosis (ALS) and frontotemporal dementia (FTD) due to the involvement of the DNA/RNA-binding proteins TDP-43 and FUS and, more recently, of C9ORF72. A potential link between dysregulation of RNA metabolism and mitochondrial dysfunction is recently emerged in TDP-43 disease models. To further investigate the possible relationship between these two pathogenetic mechanisms in ALS/FTD, we studied mitochondria functionality in human mutant *TARDBP*(p.A382T) and *C9ORF72* fibroblasts grown in galactose medium to induce a switch from a glycolytic to an oxidative metabolism. In this condition we observed significant changes in mitochondria morphology and ultrastructure in both mutant cells with a fragmented mitochondria network particularly evident in *TARDBP*(p.A382T) fibroblasts. From analysis of the mitochondrial functionality, a decrease of mitochondria membrane potential with no alterations in oxygen consumption rate emerged in *TARDBP* fibroblasts. Conversely, an increased oxygen consumption and mitochondria hyperpolarization were observed in *C9ORF72* fibroblasts in association to increased ROS and ATP content. We found evidence of autophagy/mitophagy in dynamic equilibrium with the biogenesis of novel mitochondria, particularly in mutant *C9ORF72* fibroblasts where an increase of mitochondrial DNA content and mass, and of PGC1-α protein was observed. Our imaging and biochemical data show that wild-type and mutant TDP-43 proteins do not localize at mitochondria so that the molecular mechanisms responsible for such mitochondria impairment remain to be further elucidated. For the first time our findings assess a link between *C9ORF72* and mitochondria dysfunction and indicate that mitochondria functionality is affected in *TARDBP* and *C9ORF72* fibroblasts with gene-specific features in oxidative conditions. As in neuronal metabolism mitochondria are actively used for ATP production, we speculate that *TARDBP* and *C9ORF72* mutations might trigger cell death by impairing not only RNA metabolism, but also mitochondria activity in ALS/FTD neurons.

**Electronic supplementary material:**

The online version of this article (doi:10.1186/s40478-016-0316-5) contains supplementary material, which is available to authorized users.

## Introduction

Abnormal aggregates of TDP-43 protein represent a neuropathological hallmark of the fatal motoneuron disease amyotrophic lateral sclerosis (ALS) and of fronto-temporal dementia (FTD). TDP-43 forms pathological cytoplasmic inclusions in affected brain tissues of nearly all sporadic and familial ALS cases, with the exception of *SOD1*-associated forms, and of a subset of patients with FTD (FTD-TDP), representing an important molecular link between these two apparently different neurodegenerative disorders [29]. TDP-43 is a DNA/RNA binding protein, mainly localized in the nucleus where it is involved in regulating splicing, transcription and miRNA biogenesis, but it has also regulatory functions in the cytoplasm controlling target mRNA stability, transport and translation [26]. The presence of abnormal TDP-43 aggregates in the cytoplasm together with the concurrent depletion of TDP-43 from the nucleus suggest that TDP-43 may trigger neuronal death by two different pathogenetic mechanisms including a toxic gain-of-function and/or a loss-of-nuclear function. Independently of the pathogenetic mechanism of TDP-43, RNA metabolism is strongly impaired at different levels in both ALS and FTD, as further supported by the involvement of another DNA/RNA binding protein, FUS, in the pathogenesis of these two diseases [19, 22, 36].

The more recent identification of an intronic hexanucleotide repeat expansion in *C9ORF72* gene as the major genetic cause of ALS and FTD has reinforced the molecular link between these two diseases and the role of RNA metabolism dysfunction in their pathogenesis [14, 28, 31]. The expanded *C9ORF72* transcripts, in fact, form pathological RNA foci which sequester several RNA-binding proteins and splicing factors in the nucleus, thereby globally affecting RNA processing and metabolism [15].

Mitochondrial dysfunction has long been associated with neurodegenerative diseases, including ALS, where defects in mitochondrial morphology and bioenergetics have been largely described in muscle and brain tissues from sporadic patients [1, 6, 13, 32, 33]. Moreover, changes in bioenergetics properties were recently shown in fibroblasts from sporadic ALS patients [4, 18] as well as in fibroblasts from ALS patients carrying mutations in *SOD1* and *VCP1* genes [5, 8]. In the case of *SOD1*, alterations of mitochondria functionality are directly triggered by the mutant SOD1 protein which induces mitochondrial damage [5].

Recent literature data suggest a potential link between mitochondrial dysfunction and defects in RNA metabolism associated to TDP-43 protein. Mitochondria morphology, dynamics and sub-cellular localization were described to be altered in brain tissues of TDP-43 transgenic mice as well as mitochondria functionality and transport into neurites were defective in murine neuronal cells over-expressing wild-type and mutant TDP-43 [16, 21, 34, 35, 38–40]. Localization of TDP-43 protein at mitochondria has been suggested to have a direct effect on the functionality of these organelles [16, 38].

To further assess the link between mitochondria and RNA metabolism dysfunctions, we studied mitochondria functionality in human fibroblasts of ALS and FTD patients carrying mutations in *TARDBP* and *C9ORF72* genes. In contrast to experimental disease cell models where mutant genes are over-expressed, patient-derived fibroblasts allow to better investigate pathogenetic mechanisms because mutant genes are expressed at physiological levels. As fibroblasts mainly rely on a glycolytic metabolism, they were grown in galactose medium to switch them to a mitochondrial oxidative metabolism for ATP production, similarly to neuronal cells.

## Materials and methods

### Human primary fibroblast cultures and SK-N-BE cell line

Fibroblasts were obtained from skin biopsies of 7 ALS/FTD patients carrying mutations in *TARDBP* (p.A382T, *n* = 3) and *C9ORF72* (*n* = 4) genes after informed consent and in accordance with guidelines approved by the local ethics committee (IRCCS Istituto Auxologico Italiano Review Board). Mutation analysis of *TARDBP* and *C9ORF72* genes was performed as previously described [12, 27]. Quantification of *C9ORF72* repeat expansions was obtained as reported by Akimoto et al. [3]. Clinical data are presented in Additional file 1: Table S1. Fibroblasts from 4 healthy individuals, age- and sex-matched with ALS/FTD cases, were obtained as described above (*n* = 2) and from Telethon Biobank (*n* = 2).

Fibroblasts were routinely maintained and expanded in RPMI 1640 (EuroClone, Pero, Italy) containing 2 g/l glucose and supplemented with 10 % fetal bovine serum (FBS, Sigma Aldrich, St. Louis, MO), 2 mM L-glutamine, 2,5 μg/ml amphotericin B (Sigma Aldrich), 100 units/ml penicillin and 100 μg/ml streptomycin in humid incubators at 37 °C with 5 % CO_2_. Cells used in the experiments were grown in adhesion and expanded for no more than 10 passages. To induce mitochondria to produce ATP thorough oxidative phosphorylation, fibroblasts were switched to grow for 48 h in RPMI medium deprived of glucose and containing galactose (4.5 g/l), 10 % FBS, 5 mM sodium pyruvate and 2 mM L-glutamine. All assays described, otherwise indicated, were performed in this galactose-containing medium condition.

The human neuroblastoma cell line SK-N-BE (ATCC, Middlesex, UK) was routinely maintained in RPMI 1640 supplemented with 4 g/l glucose, 10 % FBS, 2 mM L-glutamine, 1 mM sodium pyruvate, 100 units/ml penicillin and 100 μg/ml streptomycin. All reagents, otherwise indicated, were purchased from Gibco (Thermo Fisher Scientific, Waltham, MA, USA).

### Cell transfection

Fibroblasts were plated in 6-well culture plates with 12 mm diameter round coverslips and transfected with 1,5 μg/well of pDsRed2Mito vector (Clontech Laboratories, Mountain View, CA, USA) using Lipofectamine 2000 (Life Technologies, Thermo Fisher Scientific) according to the manufacturer’s instructions. After 48-h transfection, cells were fixed using 4 % paraformaldehyde in phosphate buffer saline (PBS, pH 7.4) for 15 min at room temperature. Fixed cells were used for mitochondria morphology analysis.

For mitochondria isolation, SK-N-BE cells plated in 10 mm-dishes (*n* = 10) were transfected with flagTDP-43wt or flagTDP-43p.M337V constructs (kindly provided by Dr. Wilfried Rossoll, Emory University School of Medicine, Atlanta, GA, USA) using Lipofectamine 2000. After 48- h transfection, cells were harvested for subsequent mitochondria sub-fractionation procedures.

### Mitochondrial network analysis

To visualize mitochondrial network, primary fibroblast cultures were incubated with 100 nM MitoTracker Red CMXRos (Life Technologies) for 30 min at 37 °C and then fixed with a 2:1 solution of 4 % paraformaldehyde in PBS/culture medium. Fluorescence images were acquired on Axiovert 200 epifluorescence inverted microscope (Zeiss, Oberkochen, Germany) and Shape Factor (SF) analysis was carried out using MetaMorph® software (Universal Imaging Corp., Downingtown, PA, USA). The program assigns a numeric value from 0 (flattened object) to 1 (perfect circle) to the mitochondrial morphology of a single cell. Three SF interval values were considered in our quantitative morphology analysis: group I (0–0.3) for cells with a prevalent filamentous mitochondria network; group II (0.3–0.6) for cells with a mixed population of filamentous and round-shaped mitochondria; group III (0.6–1) for cells with a prevalence of round-shaped mitochondria. A total of 40 cells for each group were analyzed.

To measure Form Factor (FF) and Aspect Ratio (AR), images of pDsRed2Mito-transfected cells were acquired with the Eclipse Ti inverted confocal microscope (Nikon, Chiyoda, Japan) and converted to a 8-bit grayscale format for analysis with ImageJ software (https://imagej.nih.gov/ij/) using two plug-ins (Adaptative threshold and isolatedpixelsremoval). Images were then converted to a binary image and areas higher than 6 pixels were analyzed. For each area the software calculates the AR parameter, defined as the ratio between the major and minor axis of the ellipse equivalent to the mitochondria, and the circularity used to obtain the FF value (1/circularity). At least 30 cells/group were considered with an average number of 80 analyzed areas/cell.

### Transmission electron microscopy

Approximately 5X10^6^ cells were washed with PBS three times and incubated with the fixing solution (glutaraldehyde 3 % in PBS 0,1 M pH 7,4) for 45 min at 4 °C protected from light. Cells were scraped and, after centrifugation at 2000 rpm at 4 °C for 15 min to form a buffy coat of cells, washed with PBS three times and post-fixed with 1 % OsO_4_ in 0,1 M PBS for 1 h. Samples were dehydrated through graded alcohols, infiltrated and embedded in SPURR resin. Ultrathin sections (60 nm) were cut with Ultrotome Nova LKB placed on copper grids and contrasted with uranyl acetate and lead citrate. Observations were performed using a ZEISS EM 109 transmission electron microscope.

### Western blot analysis

Cells were homogenized in lysis buffer (150 mM NaCl, 20 mM Tris-HCl, pH 7.4, 1 % Triton X-100, protease inhibitor cocktail) and incubated for 15 min on ice. Protein lysates (25 μg) were resolved on 4–20 % mini protean TGX Gel (Bio-Rad, Hercules, CA, USA) and transferred to nitrocellulose membrane. Immunoblots were performed with anti-MFN1 (1:1000, #ab57602), p62 (1:1000, #ab91526), p84 (1:500, #ab487) and PGC1-α (1:200, #ab77210 (Abcam, Cambridge, UK), FIS1 (1:1000, #ALX-210-1037, Enzo Life Sciences, New York, NY, USA), Tubulin (1:4000, #T6199) and TOMM20 (1;500, #WH0009804M1) (Sigma Aldrich), CASP9 (1:500, #9502), CASP3 (1:1000, #9662) and LC3 (1:1000, #2775) (Cell Signaling, Danvers, MA, USA), and TDP-43 (1:500, #10782-2-AP, Proteintech, Chicago, IL, USA) antibodies. The Clarity Western ECL Substrate (Bio-Rad) was used for chemiluminescence detection. Densitometric analyses were performed using QuantityOne software (Bio-Rad).

### Mitochondrial membrane potential

Fibroblasts were incubated with 40 nM Tetramethylrhodamine methyl ester perchlorate (TMRM; Sigma Aldrich) in HBSS-Ca/Mg solution (HEPES-buffered solution with 1.26 mM Calcium and 0.9 mM Magnesium) for 40 min at room temperature, protected from light. Labeling with the fluorescent probe MitoTracker Red CMXRos (250 nM, Life Technologies) was conducted for 30 min at 37 °C in medium protected from light. Fibroblasts were harvested with trypsin and washed twice with PBS. Cells were resuspended in FACS Flow solution (BD Biosciences, Franklin Lakes, NJ, USA) and cellular fluorescence intensity was measured using a four colours cytometer (FACSCalibur; BD Biosciences). For each sample, 10,000 events were recorded and the median of fluorescence intensity was used for the subsequent analyses with the CellQuest software (BD Biosciences).

### ATP assay

Cells were counted and 5000 cells/well were seeded in 96 white microwell plates (Thermo Fisher Scientific). Cellular ATP was measured using the ATPlite assay kit (PerkinElmer, Waltham, MA USA) according to the manufacturer’s protocol and normalized for cell number. The plate was read on the Fluoroskan Ascent FL Microplate Luminometer (Thermo Fisher Scientific) in luminescence mode.

### Oxygen consumption rate measurement

Oxygen consumption rate (OCR) was measured in adherent fibroblasts grown in galactose medium with a XF96 Extracellular Flux Analyzer (Seahorse Bioscience, North Billerica, MA, USA). Each control and mutant fibroblast cell line was seeded in a XF 96-well cell culture microplate (Seahorse Bioscience) at a density of 15 - 20 × 10^3^ cells/well in 200 μL of growth medium and incubated for 24 h at 37 °C in 5 % CO_2_ atmosphere. After replacing the growth medium with 180 μL of bicarbonate-free DMEM pre-warmed at 37 °C, cells were preincubated for 1 h before starting the assay procedure. After baseline measurements of OCR (OCR-B), OCR was measured after sequentially adding 1 μM oligomycin (OCR-O) and 2.1 μM carbonyl cyanide 4-trifluoromethoxy-phenylhydrazone (FCCP) (OCR-F). The protocol was performed as already described [17]. Data were expressed as pmol of O_2_ per minute, normalized by cell number and evaluated by the CyQUANT Cell proliferation kit (Life Technologies). Fluorescence was measured by a microplate luminometer (Victor, PerkinElmer) with excitation wavelength at 485 ± 10 nm and emission detection wavelength at 530 ± 12.5 nm.

### Mitochondrial respiratory chain enzyme activity

Enzyme activities of respiratory chain complexes were measured spectrophotometrically as described previously [10]. All enzymatic activities were normalized for citrate synthase activity and protein concentration was measured according to Lowry et al. [20].

### ROS detection

For detection of mitochondrial ROS levels, fibroblasts were incubated with 5 μM MitoSOX^TM^ (Life Technologies) in HBSS-Ca/Mg for 10 min at 37 °C. The fluorescence intensity was determined by a FACSCalibur cytometer, as described above for mitochondrial membrane potential. For cellular ROS detection, the day before the experiment 20,000 cells/well were plated in 96-well white plates and in parallel in a generic 96-well clear plate for cell counting. After incubation with 2′,7′-dichlorodihydrofluorescein (DCFH_2_) 100 μM for 30′ minutes at 37 °C in the dark, the white plate was read on a Victor plate reader (PerkinElmer), in fluorescent mode, by using appropriate filters (approximate fluorescence excitation and emission 492–495 nm/517–527 nm). Fluorescence intensity was then normalized for cell number.

### Quantitative Real time PCR

Total RNA from fibroblasts was retro-transcribed after DNaseI (Roche, Basel, Switzerland) treatment using SuperScript II-RT (Life Technologies), oligo dT and random primers. Oligonucleotide pairs for *SQSTM1/p62* and *MAP1LC3* genes were designed with Primer Express 3.0 software (Applied Biosystems, Thermo Fisher Scientific) on exon boundaries for gene expression analyses (for primer sequences see Additional file 1: Table S2). Real time PCR was performed for 45 cycles with SYBR Green PCR Master mix (Applied Biosystems) and processed on ABI Prism 7900HT (Applied Biosystems). Reactions were run in triplicate for each sample and a dissociation curve was generated at the end. Threshold cycles (C_t_) for each tested gene were normalized on the housekeeping *Rpl10a* gene value (ΔC_t_) and every experimental sample was referred to its control (ΔΔC_t_). Fold change values were expressed as 2^-ΔΔCt^.

### Mitochondrial DNA content

Total DNA was extracted from fibroblasts with the Wizard Genomic DNA Purification Kit (Promega, Fitchburg, WI, USA) according to the manufacturer’s protocol. About 20 ng of total DNA was used in real-time PCR to evaluate the mitochondria-encoded *NADH dehydrogenase 5* gene (*MT-ND5*) (Additional file 1: Table S2 for Taqman primer and probe sequences) using the genomic *ribonuclease P* (RPP) for data normalization (TaqMan RNAse P Control Reagent kit, Applied Biosystems).

### Mitochondrial mass

For determination of mitochondrial mass, fibroblasts were incubated with 75 nM Mitotracker green FM (MTG; Life Technologies) in cell medium for 30 min at 37 °C, protected from light. Cells were then harvested and processed with a FACSCalibur cytometer as described above.

### Cell viability assay

Fibroblasts were plated in 24-well dishes, in quadruplicate for each patient’s line. To determine viable/non-viable cells, both medium and adherent fibroblasts were collected. After centrifugation at 2000 rpm for 5 min, supernatant was discarded and pellet was resuspended in 20 μl medium. Cells were diluted 1:2 with trypan blue stain 0,4 % (Gibco) which labels only non-viable cells and cell viability was calculated as the number of viable cells divided by the total number of cells within a Burker’s chamber.

### Immunofluorescence

After labeling mitochondria with MitoTracker Red CMXRos dye (see above), cells were fixed as described above and permeabilized with 0.3 % Triton X-100 and blocked with 10 % normal goat serum (NGS, Gibco). Incubation with the primary antibody anti-TDP-43 (1:500, Proteintech) was performed in blocking solution for 1.30 h at 37 °C. The fluorescent-tagged secondary antibody Alexa Fluor 488 (1:500, Life Technologies) was used for detection. As a negative control, primary antibody was replaced by NGS. Nuclei were stained with 4′6-diamidino-2-phenylindole (DAPI) (Roche) and slides were mounted with Fluorsave (Calbiochem, San Diego, CA, USA). Confocal images were acquired with the Eclipse Ti inverted microscope (Nikon).

### Mitochondria sub-cellular fractionation

For mitochondria isolation 8 x 10^6^ fibroblasts (grown for 48 h in galactose-containing medium) and 20 x 10^6^ SK-N-BE cells, transfected with wild-type or mutant flagTDP-43 constructs, were pelleted at 600xg for 8 min at 4 °C, washed twice with PBS and resuspended in 1 volume of 0,1X of the hypotonic solution IB (35 mM Tris-HCl pH 7,8; 25 mM NaCl; 52 mM MgCl_2_). Cells were lysated mechanically with 20 passages through a 1 ml syringe insulin needle and 1/10 of the initial volume of packed cells of 10x IB Buffer was immediately added to make the solution isotonic. The homogenized cells were centrifuged at 1,600xg for 3 min at 4 °C and the supernatant transferred into a fresh tube. The pellet was resuspended, homogenized, isotonized and centrifuged again for twice as described above. The supernatants were added to the previous one, centrifuged at 1600xg for 3 min at 4 °C to eliminate nuclei and unbroken cells and centrifuged again at 16,000xg for 1 min. The resulting pellet, containing the mitochondria-enriched fraction, was solubilized in 1X IB Buffer and centrifuged at 16,060xg for 1 min, washed in homogenization solution A (0,32 M sucrose, 1 mM EDTA, 10 mM Tris-HCl pH 7,4) and one more time with MAITE buffer (25 mM sucrose, 75 mM sorbitol, 100 mM KCl, 0,05 mM EDTA, 5 mM MgCl_2_, 10 mM Tris-HCl pH 7,4, 10 mM H_3_PO_4_ (adjusted pH 7,4 in Tris base 0,5 M). The final pellet was resuspended in an appropriate volume of MAITE buffer. Every step was performed on ice. Total cell lysates (25 μg) and mitochondrial fractions (15 μg) were analyzed by Western blot analysis, as described above.

### Statistical analysis

Statistical analysis was conducted with PRISM software (GraphPad) by using the one-way ANOVA with Dunnett’s multiple comparison post hoc test for multiple groups analyses and the unpaired *t*-test for two-groups analyses. For the Shape Factor analysis the chi-square test was applied. For AR and FF quantitative analyses the Kruskal-Wallis with Dunns post hoc test was applied. Statistical analysis of the Seahorse data was performed using the two-way ANOVA with Bonferroni post-tests.

## Results

### Mitochondrial morphology in mTDP-43 and mC9ORF72 fibroblasts

**Fig. 1:**
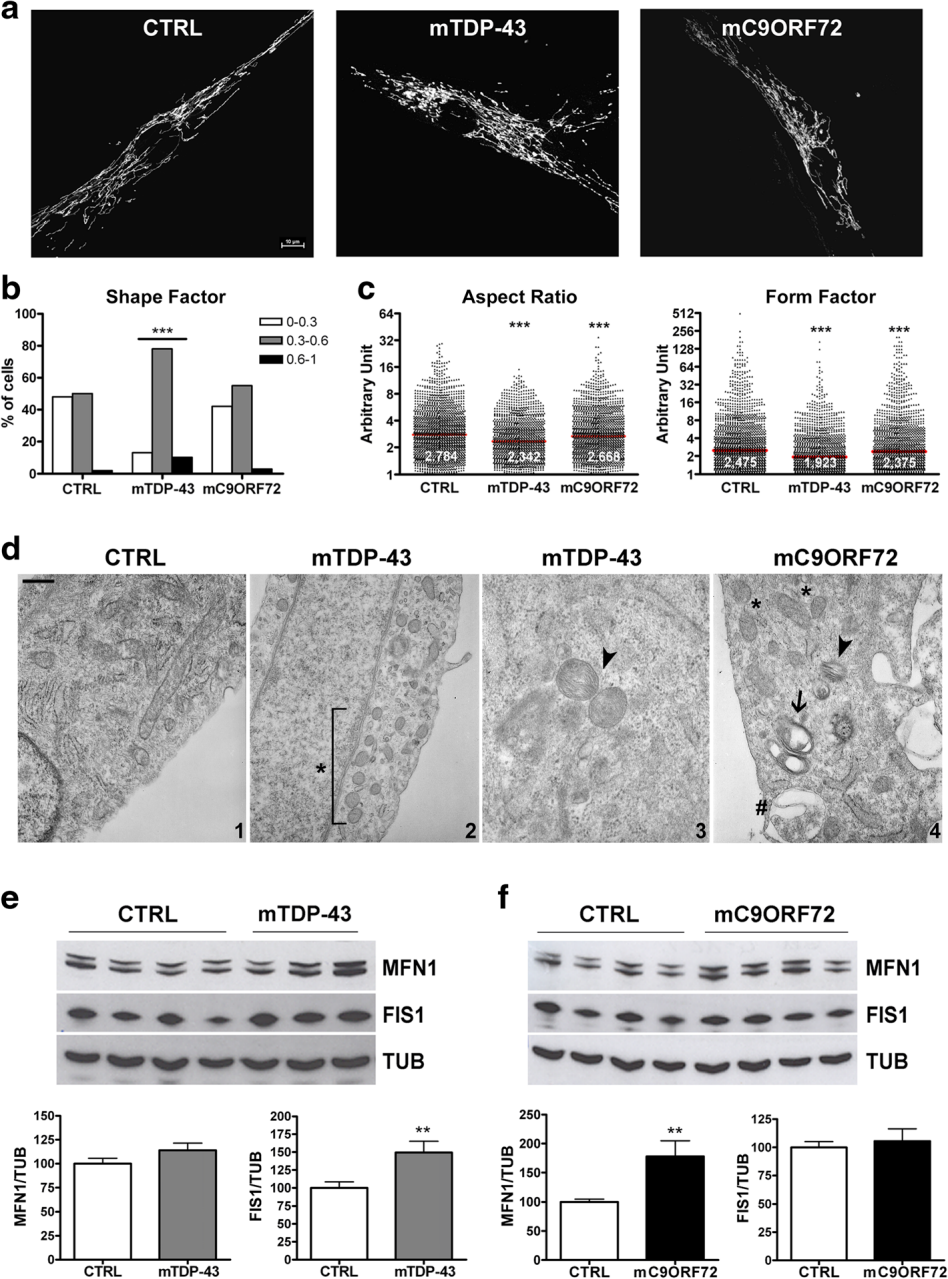
Mitochondria morphology of mutant *TARDBP* and *C9ORF72* fibroblasts after 48 h in galactose medium. **a** Representative confocal images of mitochondrial network in fibroblasts from 4 controls (CTRL), 3 ALS patients with the *TARDBP* p.A382T mutation (mTDP-43) and 4 ALS/FTD patients with mutations in *C9ORF72* (mC9ORF72). Fibroblasts were transfected with pDsRed2Mito. Bar, 10 μm. **b** Quantitative analysis of mitochondrial network in mTDP-43, mC9ORF72 and CTRL fibroblasts labelled with Mitotracker Red dye. Cell distribution in the three arbitrary chosen Shape Factor classes (0–0.3; 0.3–0.6; 0.6–1) was evaluated for each fibroblast group (mean ± SEM; Chi-square test; ***p < 0.001). **c** Quantitative analysis of mitochondrial network by Aspect Ratio (AR), an indicator of mitochondria length, and Form Factor (FF), reflecting both length and degree of mitochondria branching, in mTDP-43, mC9ORF72 and control fibroblasts transfected with pDsRed2Mito construct. Data are presented in dot plot graphs in logarithmic scale and median values are presented (red bar); Kruskal-Wallis with Dunns post hoc test; ***p < 0.001. **d** Representative images of electron microscopy analysis of CTRL (*panel 1*), mTDP-43 (*panels 2-3)* and mC9ORF72 (*panel 4*) fibroblasts. Paucity of cristae is indicated by an asterisk. A higher magnification of an unrelated mTDP-43 cell shows two degenerating mitochondria with lamellar distribution of cristae (*panel 3,* arrowhead). In mC9ORF72 fibroblasts a few autophagic vacuoles (*panel 4*, pound key) degenerating mitochondria showing mitophagic alterations (arrow) and both lamellar distribution and paucity of cristae (arrowhead) are present. Bar: 0.42 μm (*panels 1,2,4*); 0,1 μm (*panel 3*). **e** Representative WB analysis of MFN1 and FIS1 proteins in CTRL and mTDP-43 fibroblasts (*upper panel*). Densitometric analyses of WB data are shown (mean ± SEM of 4 controls and 3 *TARDBP* p.A382T patients, unpaired *t*-test, **p < 0.01) (*lower panel*). Lysates from 3 different DIV were considered for each cell line. **f** Representative WB (*upper panel*) and densitometric analyses (*lower panel*) of CTRL and mC9ORF72 fibroblasts (mean ± SEM; *n* = 3 different *DIV*; unpaired *t*-test, **p < 0.01)

To evaluate mitochondrial functionality in fibroblasts of ALS/FTD patients carrying pathogenic mutations in *TARDBP* and *C9ORF72* genes, we first analyzed mitochondria morphology and network organization as potential indicators of their pathophysiology. As fibroblasts have a mainly glycolytic metabolism, they were grown in oxidative conditions in galactose-containing medium for 48 h to induce them to rely on oxidative phosphorylation (OX-PHOS) for energy production [30]. Primary fibroblasts obtained from 3 ALS patients harboring the same mutation (p.A382T) in *TARDBP* gene, 4 patients with pathological expansion of the hexanucleotide repeat sequence in *C9ORF72* gene and 4 healthy controls (Additional file 1: Table S1) were transfected with the mitochondria-tagging plasmid pDsRed2Mito or live-labeled with the Mitotracker Red dye. In these oxidative conditions mutant TDP-43 (mTDP-43) fibroblasts showed fragmentation of the mitochondria network compared to control cells, with the prevalence of round-shaped mitochondria (Fig. 1a and Additional file 1: Figure S1). Mutant C9ORF72 (mC9ORF72) fibroblasts showed a less evident alteration of mitochondria network compared to mTDP-43 cells, but mixed populations of elongated, short and round-shaped mitochondria were frequently observed within a single cell (Fig. 1a and Additional file 1: Figure S1). In contrast, no morphological alterations of mitochondria were observed in mutant fibroblasts maintained in standard growing conditions with glucose as a main carbon source (data not shown).

For the quantitative analysis of the mitochondrial network changes observed in oxidative condition, we measured the Shape Factor (SF) parameter, an index of cell mitochondria morphology and organization with values ranging from 0 (cells with flattened mitochondria) to 1 (cells with circular mitochondria). Three main categories were arbitrary chosen and cell distribution in the three different classes (0-0.3; 0.3–0.6; 0.6–1) was evaluated for each fibroblast group (Fig. 1b and Methods). The pattern of cell distribution was significantly different in mTDP-43 fibroblasts compared to controls. In particular, a decreased number of cells in the first category (0–0.3) and an increased amount of cells in the other two categories was evident, indicating fragmentation of the mitochondria network (Fig. 1b). No significant differences were detected in mC9ORF72 fibroblasts compared to controls, although a slight increase of the intermediate class was observed (Fig. 1b).

A more detailed image analysis was performed on single mitochondria area to measure other two quantitative morphological parameters, such as Aspect Ratio (AR), an indicator of mitochondria length, and Form Factor (FF), reflecting both length and degree of mitochondria branching. Both AR and FF values were significantly lower in both mTDP-43 and mC9ORF72 fibroblasts compared to healthy cells, although differences were higher for mTDP-43 mitochondria (Fig. 1c). Quantitative morphology analyses clearly suggest that mitochondria network is altered in both mutant fibroblast groups, with mTDP-43 cells presenting a more fragmented mitochondria network compared to mC9ORF72.

In line with these results, electronic microscopy analysis indicated that, although both mutant fibroblasts had a normal aspect considering the reticulum, membranes and nuclei, they showed some mitochondria alterations in all samples examined, namely lamellar distribution and paucity of cristae (Fig. 1d). Few autophagic and mitophagic vacuoles were also present in both mTDP-43 and mC9ORF72 cells (Fig. 1d).

Mitochondria dynamics was then investigated by assessing the content of two factors involved in fission and fusion processes, such as Fission 1 (FIS1) and Mitofusin 1 (MFN1) proteins, respectively. Western blot (WB) analyses indicated that FIS1 levels were significantly increased in mTDP-43 fibroblasts (149 %) compared to control cells (Fig. 1e), while MFN1 content was increased specifically in mC9ORF72 cells (178 %) (Fig. 1f). These results suggest that an altered balance of fission and fusion processes may account for the mitochondria morphology changes observed in mTDP-43 and mC9ORF72 fibroblasts.

### Mitochondrial functionality of mTDP-43 and mC9ORF72 fibroblasts

**Fig. 2:**
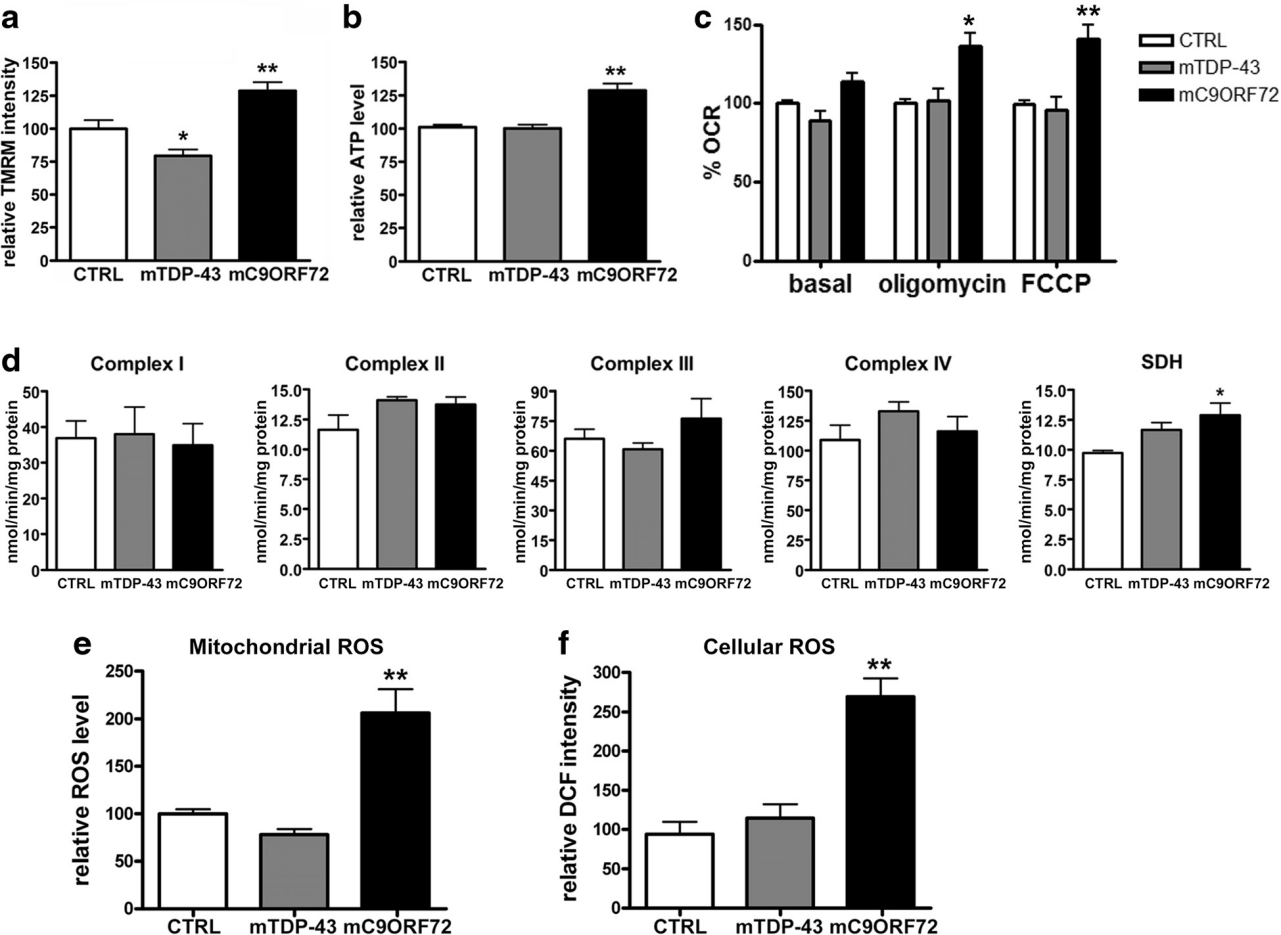
Mitochondrial functionality of mutant *TARDBP* and *C9ORF72* fibroblasts in galactose medium. **a** Measurement of mitochondrial membrane potential (MMP) by flow cytometry analysis of TMRM-positive fibroblasts (4 healthy controls, 3 *TARDBP* p.A382T (mTDP-43) and 4 *C9ORF72* (mC9ORF72). Median ± SEM, *n* = 3 different *DIV*; one-way ANOVA with Dunnett’s multiple comparison test, *p < 0.05; **p < 0.01. **b** Evaluation of ATP content by the ATPlite kit. Mean ± SEM, *n* = 3 different *DIV*; one-way ANOVA with Dunnett’s multiple comparison test, **p < 0.01. **c.** Analysis of oxygen consumption rate in basal condition (OCR-B), upon treatment with oligomycin (OCR-O) and with the oxidative phosphorylation uncoupler FCCP (OCR-F). Data are presented as mean ± SEM; *n* = 5 different *DIV*; two-way ANOVA with Bonferroni post-tests; *p < 0.05 and **p < 0.01 vs. basal. **d** Measurement of the respiratory electron transport system activity (complexes I-IV) and of succinate dehydrogenase (SDH) in 3 CTRL, 3 mTDP-43 and 4 mC9ORF72 fibroblasts. Values are expressed as nmol/min/mg protein and normalised with citrate synthase activity (mean ± SEM, one-way ANOVA with Dunnett’s multiple comparison test; *p < 0.05). **e** Mitochondrial ROS detection by flow cytometry analysis after incubation with MitoSOX™ Red reagent. Mean ± SEM; *n* = 3 different *DIV*; one-way ANOVA with Dunnett’s multiple comparison test; **p < 0.01. **f** Cellular ROS detection after incubation with 2′,7′-dichlorodihydrofluorescein (DCF). Fluorescence intensity was normalized for cell number. Mean ± SEM; *n* = 3 different *DIV*; one-way ANOVA with Dunnett’s multiple comparison test; **p < 0.01

To investigate the potential correlation between the mitochondria morphological changes observed in both mutant fibroblasts in oxidative state and their bioenergetic efficiency, we measured mitochondrial membrane potential (MMP) with TMRM dye by FACS analysis. We observed a significant depolarization of mitochondria in mTDP-43 fibroblasts, where the median MMP value was approximately 80 % compared to control group, whereas in mC9ORF72 fibroblasts the median MMP value significantly increased to 129 %, indicating hyperpolarized mitochondria (Fig. 2a). These results were further confirmed by measuring MMP with another MMP-dependent dye, the MitoTracker Red CMXRos (Additional file 1: Figure S2). No changes in MMP were instead detected when fibroblasts were grown in glycolytic conditions (Additional file 1: Figure S3), reflecting the absence of morphological alterations of mitochondria in the same conditions.

In line with the increase of MMP value in mC9ORF72 fibroblasts, a significant increase of ATP content was observed, whereas no change of ATP levels was found in mTDP-43 cells (Fig. 2b). To further characterize mitochondrial functionality in oxidative state, we evaluated oxygen consumption rate (OCR) in control and mutant fibroblasts in different conditions: 1) in basal conditions (OCR-B); 2) when oxygen consumption was inhibited by the ATP synthesis blocker oligomycin (OCR-O); 3) when oxygen consumption was stimulated by the oxidative phosphorylation uncoupler FCCP (OCR-F). We observed that the overall oxygen consumption rate was enhanced in mC9ORF72 fibroblasts, with both OCR-O and OCR-F reaching statistically significant increase as compared to both controls and mTDP-43 cells (Fig. 2c), indicating high activity of the electron transport chain (ETC).

Since global mitochondria respiration emerged to be altered specifically in mC9ORF72 fibroblasts, the biochemical analysis of each single ETC complex activity was performed. No significant differences were measured in both mutant fibroblasts compared to controls, although for some complexes, we noticed a tendency toward an increase (Fig. 2d and Additional file 1: Table S3). We observed that succinate dehydrogenase (SDH) activity was significantly higher in mC9ORF72 fibroblasts compared to control cells and a trend toward an increase was also present in mTDP-43 fibroblasts although it did not reach statistical significance (Fig. 2d).

In order to investigate if the mitochondria bioenergetic dysfunctions were associated with oxidative stress, we next measured reactive oxygen species (ROS) levels in mutant cells. In particular, we evaluated total cellular ROS levels by DCFH_2_ reagent and, as the results of such measurement may be influenced by other factors [9], specific mitochondrial ROS levels were determined by using mitoSOX. We found a consistent and significant increase of mitochondrial ROS along with a parallel increase of total cellular ROS levels specifically in mC9ORF72 cells compared to both healthy control and mTDP-43 fibroblasts (Fig. 2e-f).

### The mitochondria quality system in mTDP-43 and mC9ORF72 fibroblasts

**Fig. 3:**
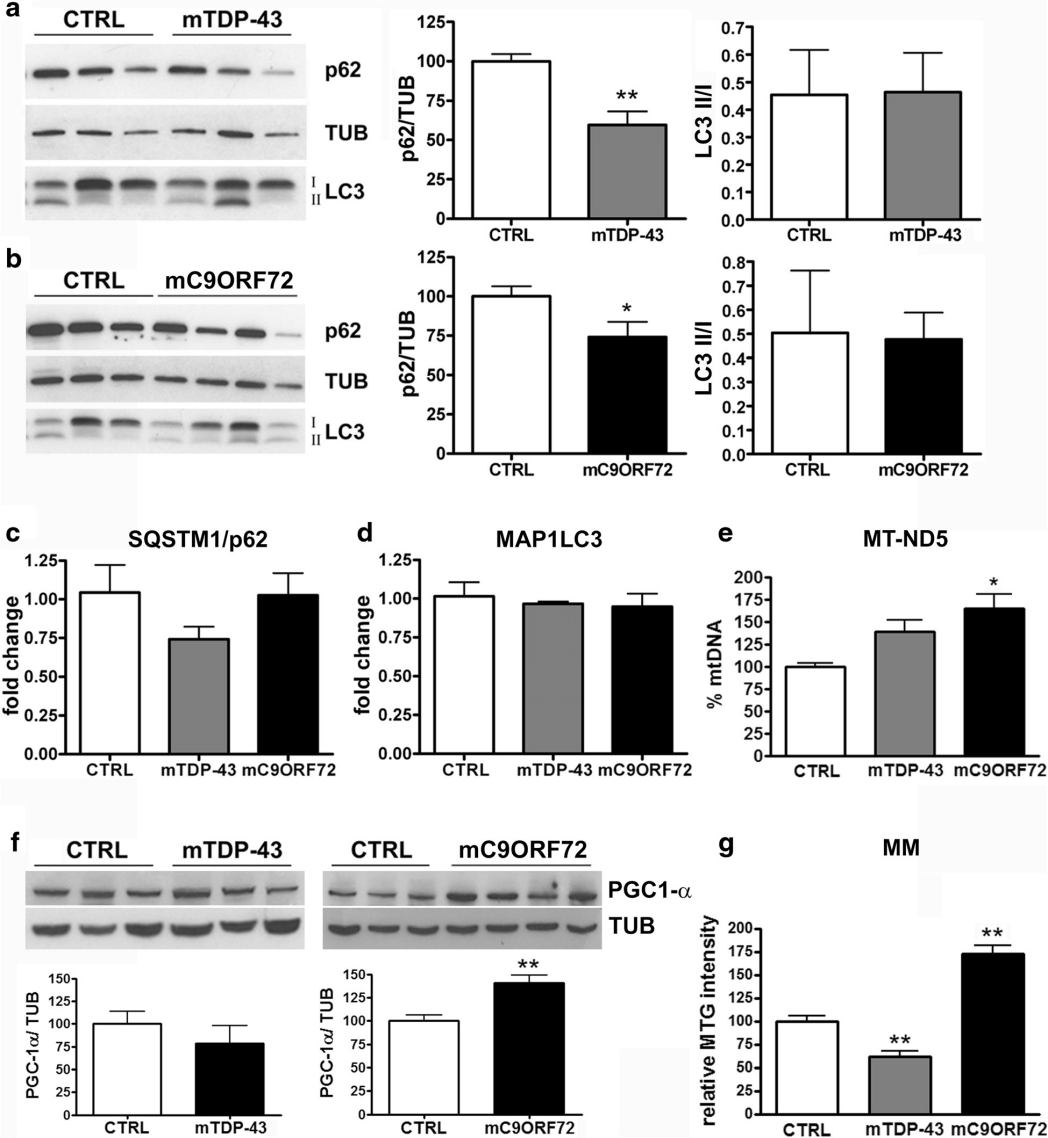
Analysis of autophagy and mitochondria biogenesis in mutant *TARDBP* and *C9ORF72* fibroblasts in galactose medium. **a** Representative WB analysis of p62 and LC3II/I in lysates of control (CTRL) and mutated *TARDBP* (mTDP-43) fibroblasts (*left panel*). Tubulin was used for sample normalization. Densitometric analyses of WB data are shown in the *right* panels; the ratio between lipidated (II) and unlipidated (I) protein is shown for LC3 (mean ± SEM of 3 controls and 3 mTDP-43 patients; *n* = 3 different *DIV*; unpaired *t*-test, **p < 0.01). **b** Representative WB analysis of p62 and LC3II/I in lysates of CTRL and mutated C9ORF72 (mC9ORF72) fibroblasts (*left panel*). Tubulin was used for sample normalization. Densitometric analyses of WB data are shown in *right* panels (mean ± SEM of 3 controls and 4 mC9ORF72 patients, *n* = 3 different *DIV*; unpaired *t*-test, *p < 0.05). Q-PCR analysis of **c** *SQSTM1/p62* and **d** *MAP1LC3* mRNA content in CTRL, mTDP-43 and mC9ORF72 fibroblasts. Fold change values were calculated versus CTRL fibroblasts (mean ± SEM; *n* = 3 different *DIV*; one-way ANOVA with Dunnett’s multiple comparison test). **e** Quantification of mitochondrial DNA content by Q-PCR of the mitochondria-encoded *NADH dehydrogenase 5* (*MT-ND5*) gene. The genomic *ribonuclease P* gene was used for data normalization (mean ± SEM; *n* = 3 for each experimental group; one-way ANOVA with Dunnett’s multiple comparison test). **f** Representative WB analysis of PGC1α in lysates of controls (CTRL), mTDP-43 (*left panel*) and mC9ORF72 (*right panel*) fibroblasts. Tubulin was used for sample normalization. Densitometric analyses of WB data are shown in the *lower left* and *right panels* (mean ± SEM; *n* = 3 different *DIV*; unpaired *t*-test, *p < 0.05). **g** Measurement of mitochondrial mass (MM) by flow cytometry analysis of Mitotracker green (MTG)-positive fibroblasts (4 healthy controls, 3 mTDP-43 and 4 mC9ORF72). Median ± SEM; *n* = 3 different *DIV*; one-way ANOVA with Dunnett’s multiple comparison test; **p < 0.01

Given the alterations in mitochondrial morphology and functionality observed in mTDP-43 and mC9ORF72 fibroblasts, we next investigated the processes associated to removal of damaged mitochondria and/or biogenesis of these organelles. Quantification of p62 and LC3 levels, as markers of autophagic vesicle formation, showed a significant decrease of p62 protein level both in mTDP-43 (Fig. 3a) and in mC9ORF72 fibroblasts (Fig. 3b) (60 and 74 %, respectively) compared to control fibroblasts with no change in *SQSTM1/p62* gene expression level (Fig. 3c). The ratio between lipidated/unlipidated LC3 proteins (LC3-II/LC3-I) and the total *MAP1LC3B* mRNA content were not different between mutant fibroblasts and control cells (Figs. 3a-b-d). These results, together with electronic microscopy data showing few autophagic/mitophagic vacuoles in both mutant fibroblast groups (Fig. 1d), suggest an early activation of the autophagic process.

As removal of damaged mitochondria is a dynamic process in equilibrium with the biogenesis of novel organelles, we measured the content of mitochondrial DNA as a marker of mitochondrial biogenesis. The level of mitochondria-encoded *NADH dehydrogenase 5* (*MT-ND5*) was slightly increased in mTDP-43 fibroblasts, but significantly higher in mC9ORF72 fibroblasts as compared to controls (Fig. 3e). Moreover, evaluation of PGC1-α protein, a regulator of mitochondria biogenesis, showed significantly increased levels only in mC9ORF72 fibroblasts, while no changes in mTDP-43 fibroblasts were detected compared to controls (Fig. 3f). Differences in markers of mitochondria biogenesis in mutant mC9ORF72 fibroblasts reflected also differences in mitochondria content. In fact, mitochondria mass (MM), measured by Mitotracker GreenFM dye, significantly and specifically increased in mC9ORF72 fibroblasts (172 %) compared to control cells, whereas it significantly diminished in mTDP-43 cells (65 %) (Fig. 3g). No changes in MM were observed in mutant fibroblasts grown in standard culture conditions in glucose-containing medium (Additional file 1: Figure S4), further suggesting that mitochondria alterations become evident only when cells are forced to switch from a glycolytic to an oxidative state.

### Cell viability and apoptosis in mTDP-43 and mC9ORF72 fibroblasts

**Fig. 4:**
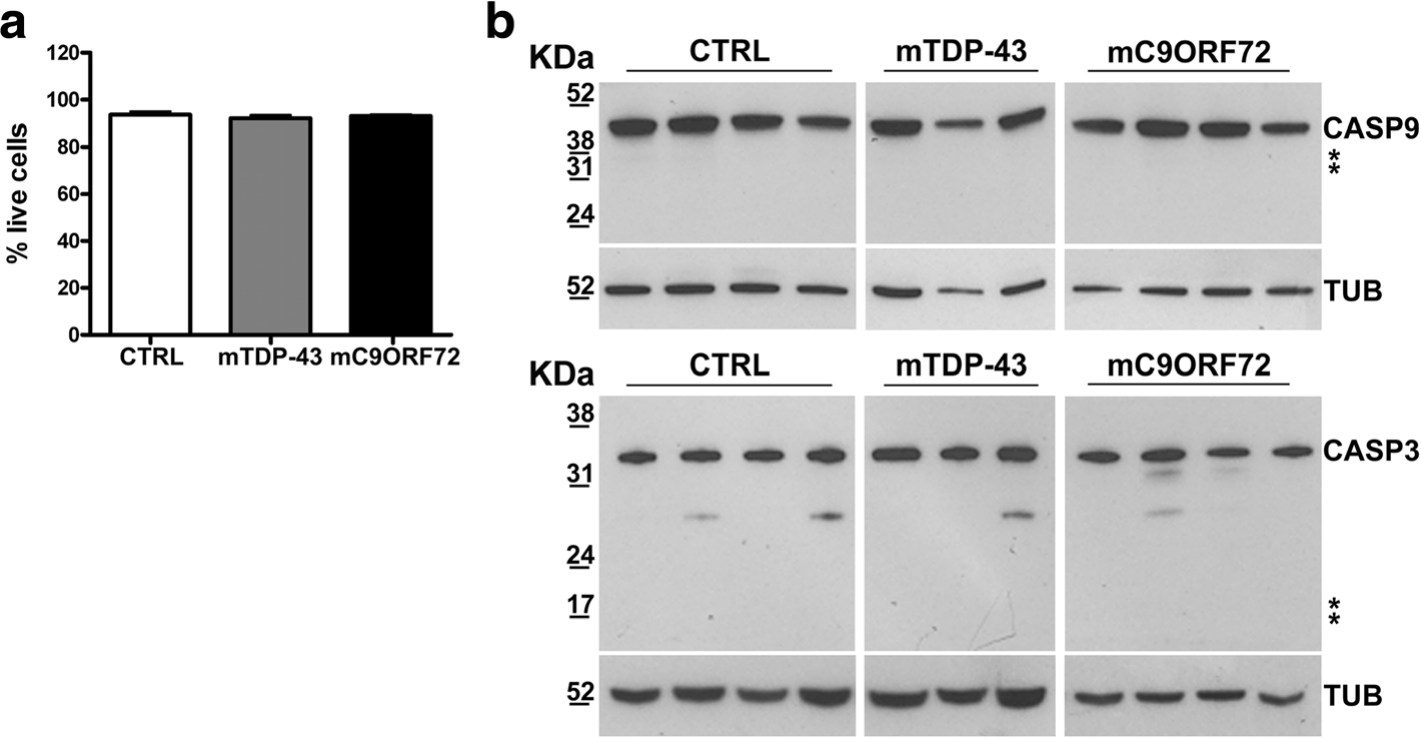
Cell viability and apoptosis in mutant *TARDBP* and *C9ORF72* fibroblasts in galactose medium. **a** Cell viability was assessed by trypan blue stain in control (CTRL), mutated *TARDBP* p.A382T (mTDP-43) and *C9ORF72* (mC9ORF72) fibroblasts. **b** WB analysis of caspase 9 (CASP9) and 3 (CASP3) in lysates of 4 CTRL, 3 mTDP-43 and 4 mC9ORF72 fibroblasts. *Asterisk* indicates the expected position of the cleaved forms of the two caspases. *n* = 3 different *DIV*

To evaluate if the observed alterations in autophagy and mitochondria biogenesis may trigger cell death, we measured cell viability and we found no evidence of cell mortality in both mTDP-43 and mC9ORF72 fibroblasts compared to control cells (Fig. 4a). Evaluation of Caspase 9 and Caspase 3 by WB assay revealed no changes in their protein pattern in both control and mutant fibroblasts indicating no activation of the apoptotic pathway (Fig. 4b).

### Analysis of TDP-43 sub-cellular localization

Recent literature data on mouse motoneuronal cell lines and primary neurons reported that both wild-type and mutant TDP-43 proteins may localize at mitochondria thereby directly affecting their functionality [16, 21, 38]. We investigated whether the endogenous wild-type and mutant TDP-43 distributed at mitochondria also in human fibroblasts.

**Fig. 5:**
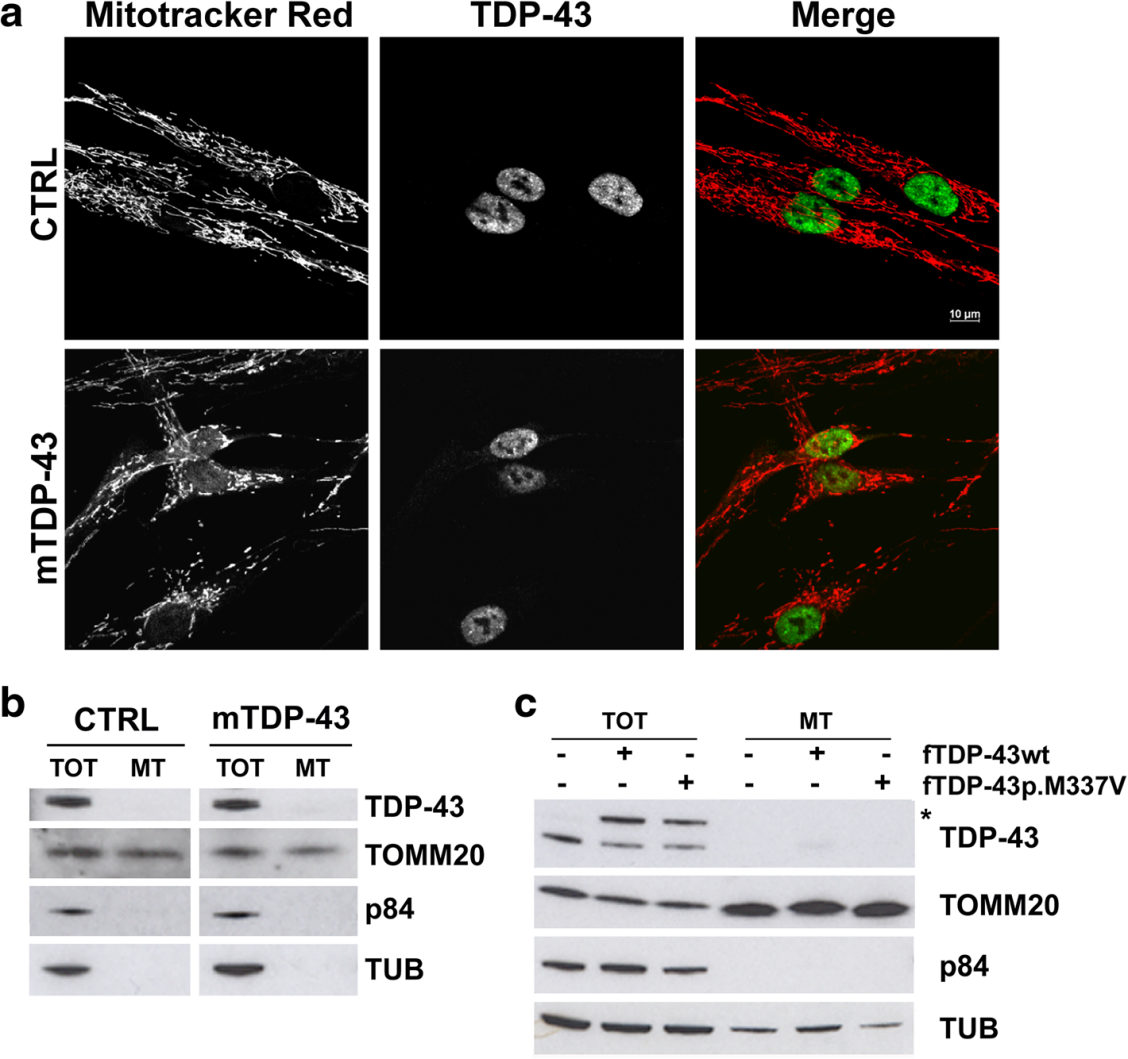
Analysis of TDP-43 sub-cellular localization in human fibroblasts and SKNBE cells. **a** Representative immunofluorescence images of TDP-43 (green) in primary fibroblasts from 4 healthy controls (CTRL) and 3 ALS patients carrying p.A382T mutation in *TARDBP* gene (mTDP-43), incubated with Mitotracker Red (red). Bar, 10 μm. **b** WB analysis of TDP-43 and TOMM20 proteins in total cell lysates (TOT) and mitochondria-enriched fractions (MT) of CTRL and mTDP-43 fibroblasts maintained in galactose medium for 48 h. p84 and tubulin (TUB) were used to test contamination with nuclear and cytoplasmic fractions, respectively. **c** WB analysis of TDP-43 and TOMM20 proteins in total cell lysates (TOT) and mitochondria-enriched fractions (MT) of SK-N-BE cells transfected for 48 h with flag-tagged wild-type (fTDP-43wt) and mutant p.M337V (fTDP-43p.M337V) TDP-43 constructs. *Asterisk* indicates flag-tagged TDP-43 proteins. p84 and tubulin (TUB) were used as nuclear and cytoplasmic markers, respectively. Different protein amounts were loaded for TOT (25 μg) and MT (15 μg) fractions

By immunofluorescence analysis we failed to detect TDP-43 co-localization with mitochondria and TDP-43 mislocalization in the cytoplasm both in control and mTDP-43 fibroblasts (Fig. 5a). Also in mC9ORF72 cells no localization of TDP-43 at mitochondria was observed (Additional file 1: Figure S5). By performing sub-cellular fractionation assays in control and mTDP-43 fibroblasts, both wild-type and mutant TDP-43 proteins were not present in the mitochondria-enriched fractions, which were positive for the mitochondria marker TOMM20 (Fig. 5b). Nuclear and cytoplasmic contamination of the obtained mitochondria-enriched fractions was evaluated by WB analysis with p84 and tubulin markers, respectively (Fig. 5b). When we also over-expressed wild-type and mutant TDP-43 p.M337V in human neuroblastoma SK-N-BE cells, we did not recover either proteins in the mitochondria-enriched fractions (Fig. 5c), in contrast to what previously reported [16].

In addition, an *in silico* analysis of TDP-43 aminoacid sequence by Mitoprot, PSORT, and TargetP algorithms failed to recognize the presence of the consensus motif for protein import into mitochondria.

Overall these data seem to suggest that the mitochondria dysfunctions observed in mTDP-43 fibroblasts are not likely to be directly associated with localization of TDP-43 protein at mitochondria.

## Discussion

In the present study we showed that mutations in *TARDBP* and *C9ORF72* genes, associated to defects in RNA metabolism in ALS and FTD neurodegenerative diseases, affect mitochondria functionality by altering several morphological and bioenergetics parameters in a gene-specific manner. These alterations were detected in peripheral disease cell models derived from patients, such as mutant fibroblasts, when they were induced to change their glycolytic metabolism and to actively use oxidative phosphorylation for ATP production as neuronal cells.

In neurodegenerative disorders the study of pathomechanisms is hampered by the difficulty to monitor disease progression in the affected brain tissues, and autoptic tissues have the bias of representing the end-stage state of the disease. Nonetheless, in Alzheimer’s and Parkinson’s disorders, pathophysiological alterations of the central nervous system were also similarly present in patients’ peripheral tissues, such as blood and fibroblasts [7, 24]. Although fibroblasts have been scarcely investigated in ALS disease so far [2, 23], they were recently described to be defective in their bioenergetics metabolism both in familial and sporadic ALS cases [5, 8, 18, 25]. In contrast to experimental disease cell models where the mutant genes are usually over-expressed, patient-derived fibroblasts have the advantage of expressing mutant genes at physiological levels, therefore preventing any bias due to the aberrant over-production of the protein of interest.

Our analysis of mitochondrial functionality in mTDP-43 and mC9ORF72 fibroblasts revealed gene-specific alterations in mitochondrial morphology and dynamics, membrane potential, respiration and ATP production specifically occurring in oxidative conditions. In fact, these changes were absent when mutant fibroblasts were grown in glycolytic conditions. We found alterations in mitochondria morphology in both mTDP-43 and mC9ORF72 fibroblasts, as indicated by significant changes in AR and FF quantitative parameters and in mitochondrial ultrastructure and density by electronic microscopy. In particular, a very fragmented mitochondria network was evident in mTDP-43 fibroblasts in association to the up-regulation of FIS1 protein content. An altered balance of fission and fusion processes, also evidenced in mC9ORF72 fibroblasts with increased MFN1 levels, may well account for the observed mitochondria morphology changes. Interestingly, the presence of altered mitochondria with paucity of cristae was previously described in spinal cord of TDP-43 transgenic mice in association with an abnormal sub-cellular distribution and trafficking of mitochondria and with dysregulation of proteins involved in fission and fusion processes [34, 35, 38–40]. Also murine neuronal cell models over-expressing wild-type or mutant human TDP-43 proteins were described to have alterations in mitochondria morphology, dynamics and transport along neurites [16, 21, 38]. On the other hand, mitochondria alterations have not been reported or investigated in C9ORF72 cellular and animal models so far, but our findings suggest that C9ORF72-mediated pathomechanisms, either through expanded RNA/dipeptide protein gain-of-function or C9ORF72 protein haploinsufficiency, may also affect mitochondria metabolism. Our data also suggest that mutant *TARDBP* and *C9ORF72* trigger mitochondria alterations in response to energy demand in a specific manner because mitochondrial morphological changes were not present in mutant *SOD1* and sporadic ALS fibroblasts when they were similarly exposed to oxidative conditions in galactose-containing medium [4, 5].

In line with the evidence that mitochondria morphology may reflect mitochondria functionality, we found alterations in the mitochondria membrane potential, which significantly changed in a different manner in the two mutant fibroblasts. Mitochondria membrane potential was specifically reduced in mTDP-43 fibroblasts and increased in mC9ORF72 cells. Mitochondria depolarization was also described in murine TDP-43 cell models, but when both wild-type and mutant TDP-43 were over-expressed in primary motor neurons or in NSC-34 cells [16, 21, 38]. Human fibroblasts from sporadic ALS patients showed mitochondria hyperpolarization when they were grown in glycolytic conditions [18]. Here we found that mutations in *TARDBP* and *C9ORF72* were associated to alterations of mitochondria membrane potential only when cells had to rely mainly on oxidative phosphorylation for ATP synthesis. When we evaluated the mitochondria bioenergetics, we observed an increase of oxygen consumption rate only in mC9ORF72 fibroblasts after blocking ATP synthesis and after uncoupling respiration, in association with normal respiratory chain enzymatic activities but increased SDH activity. A similar increase in oxygen consumption was observed also in mutant *SOD1* fibroblasts when they were exposed to galactose-containing growing medium, although this was not associated with mitochondria morphological alterations as in mC9ORF72 cells [5]. The increased oxygen consumption rate may reflect an efficient and tightly coupled ETC activity in mC9ORF72 fibroblasts, thus accounting for the observed higher ATP content, while the increased MMP may favour electron slippage and trigger ROS production as sustained by our experimental data.

Among the mitochondrial changes, we found also an increase of mitochondrial DNA content and mass specifically in mC9ORF72 fibroblasts, suggesting the presence of compensatory proliferation mechanisms of aberrant mitochondria, which could also explain the higher oxygen consumption rate, ATP level and ROS production in these cells. We speculate that the same mechanism is probably not active in mTDP-43 fibroblasts which showed depolarized mitochondria, but were not able to activate mitochondrial proliferation to counteract mitochondrial dysfunctions. Since mitochondrial fission usually precedes mitophagy [11, 37], the observed fragmentation of the mitochondrial network and the presence of autophagic/mitophagic vacuoles in mTDP43 fibroblasts seem to support this hypothesis. The autophagic process is also partially activated in mC9ORF72 as evidenced by electron microscopy data and p62 protein down-regulation.

Whether a direct or an indirect effect of mTDP-43 and mC9ORF72 is responsible for the observed mitochondria dysfunctions still remains to be elucidated. Our data suggest that in human fibroblasts both wild-type and mutant TDP-43 proteins do not localize at mitochondria and therefore are not likely to influence directly their functioning. Also in condition of TDP-43 over-expression in human neuronal cell lines, we did not observe translocation of TDP-43 into mitochondria. On the contrary, recent literature data supported the hypothesis of a direct effect on mitochondria based mainly on the presence of TDP-43 protein in mitochondria-enriched fractions obtained by cell fractionation assays [16, 38]. However, as contamination with nuclear materials, and likely nuclear TDP-43, may not be completely excluded in these assays, this issue will need further investigation.

Altogether our results indicate that mutations in *TARDBP* and *C9ORF72* genes may differentially affect mitochondria activity and bioenergetics in ALS and FTD patient-derived cells when they are induced to use oxidative phosphorylation for ATP production. This further supports the evidence of a link between defects in TDP-43-mediated RNA metabolism and mitochondria dysfunction [16, 21, 38]. However, as in this study we considered ALS patients carrying the same *TARDBP* p.A382T mutation, which is the most frequent one in the Italian population [12], also different *TARDBP* mutations will worth investigation.

## Conclusions

Our findings highlight for the first time that mutant C9ORF72 impairs mitochondria activity in a different and opposite manner compared to mutant TDP-43. We speculate that TARDBP and C9ORF72 mutations might trigger cell death by impairing not only RNA metabolism, but also mitochondria activity in ALS/FTD neurons since their mitochondrial energetic metabolism depends mainly on oxidative phosphorylation. The use of neurons/motoneurons differentiated from induced pluripotent stem cells obtained from mTDP-43 and mC9ORF72 fibroblasts will certainly help to address and further investigate this important issue.

## Additional file

Additional file 1: Figure S1. Mitochondria morphology in mutant *TARDBP* and *C9ORF72* fibroblasts after 48 h in galactose medium. Representative confocal images of mitochondrial network in primary fibroblasts from three healthy controls (C1, C2, C3), three ALS patients carrying mutation in *TARDBP* gene (p.A382T; T1, T2, T3) and three ALS//FTD patients with pathological expansion of the hexanucleotide repeat in *C9ORF72* (C9.2, C9.3, C9.4) transfected with pDsRed2Mito construct. Bar, 10μm. **Figure S2.** Mitochondria functionality in mutant *TARDBP* and *C9ORF72* fibroblasts. Measurement of mitochondrial membrane potential by flow cytometry analysis of Mitotracker Red (MTR) positive fibroblasts (from 4 healthy controls, 3 *TARDBP* and 4 *C9ORF72* mutated patients) maintained in medium with galactose and without glucose. Median ± SEM, *n* = 3 different *DIV*; One-way ANOVA with Dunnett’s multiple comparison test; ***p* < 0.01). **Figure S3.** Analysis of mitochondria membrane potential in mutant *TARDBP* and *C9ORF72* fibroblasts in glycolitic conditions. Measurement of mitochondrial (MMP) by flow cytometry analysis of TMRM-positive fibroblasts (4 healthy controls, 3 *TARDBP* and 4 *C9ORF72*), maintained in medium with 2 g/l glucose. Median ± SEM, *n* = 3 different *DIV*; One-way ANOVA with Dunnett’s multiple comparison test). **Figure S4.** Analysis of mitochondria mass in mutant *TARDBP* and *C9ORF72* fibroblasts in glycolitic conditions. Measurement of mitochondrial mass (MM) by flow cytometry analysis of Mitotracker green (MTG)-positive fibroblasts (4 healthy controls, 3 *TARDBP* and 4 *C9ORF72*), maintained in medium with 2 g/l glucose. Median ± SEM, *n* = 3 different *DIV*; One-way ANOVA with Dunnett’s multiple comparison test). **Figure S5.** TDP-43 sub-cellular localization in *C9ORF72* fibroblasts after 48 h in galactose medium. Representative immunofluorescence images of TDP-43 (green) in primary fibroblasts from healthy controls (four) and ALS/FTD patients carrying mutation in *C9ORF72* gene (four), incubated with Mitotracker Red. Bar, 10 μm. **Table S1.** Clinical features of healthy controls, *TARDBP-* and *C9ORF72*-mutated patients. **Table S2.** Primer sequences and probes for Quantitative Real time PCR. **Table S3.** Respiratory electron transport chain activity. Values are expressed as nmol/min/mg protein and normalised with citrate synthase activity. (PDF 609 kb)
