# Supplementary material for: Gene-specific mitochondria dysfunctions in human TARDBP and C9ORF72 fibroblasts
Source: Acta Neuropathol Commun. 2016 May 5;4:47. doi: 10.1186/s40478-016-0316-5 (PMC4858818; doi:10.1186/s40478-016-0316-5)
Supplement: Additional file 1: Figure S1. — Mitochondria morphology in mutant TARDBP and C9ORF72 fibroblasts after 48 h in galactose medium. Representative confocal images of mitochondrial network in primary fibroblasts from three healthy controls (C1, C2, C3), three ALS patients carrying mutation in TARDBP gene (p.A382T; T1, T2, T3) and three ALS//FTD patients with pathological expansion of the hexanucleotide repeat in C9ORF72 (C9.2, C9.3, C9.4) transfected with pDsRed2Mito construct. Bar, 10μm. Figure S2. Mitochondria functionality in mutant TARDBP and C9ORF72 fibroblasts. Measurement of mitochondrial membrane potential by flow cytometry analysis of Mitotracker Red (MTR) positive fibroblasts (from 4 healthy controls, 3 TARDBP and 4 C9ORF72 mutated patients) maintained in medium with galactose and without glucose. Median ± SEM, n = 3 different DIV; One-way ANOVA with Dunnett’s multiple comparison test; **p < 0.01). Figure S3. Analysis of mitochondria membrane potential in mutant TARDBP and C9ORF72 fibroblasts in glycolitic conditions. Measurement of mitochondrial (MMP) by flow cytometry analysis of TMRM-positive fibroblasts (4 healthy controls, 3 TARDBP and 4 C9ORF72), maintained in medium with 2 g/l glucose. Median ± SEM, n = 3 different DIV; One-way ANOVA with Dunnett’s multiple comparison test). Figure S4. Analysis of mitochondria mass in mutant TARDBP and C9ORF72 fibroblasts in glycolitic conditions. Measurement of mitochondrial mass (MM) by flow cytometry analysis of Mitotracker green (MTG)-positive fibroblasts (4 healthy controls, 3 TARDBP and 4 C9ORF72), maintained in medium with 2 g/l glucose. Median ± SEM, n = 3 different DIV; One-way ANOVA with Dunnett’s multiple comparison test). Figure S5. TDP-43 sub-cellular localization in C9ORF72 fibroblasts after 48 h in galactose medium. Representative immunofluorescence images of TDP-43 (green) in primary fibroblasts from healthy controls (four) and ALS/FTD patients carrying mutation in C9ORF72 gene (four), incubated with Mitotracker Red. Bar, 10 μm. Table [file 40478_2016_316_MOESM1_ESM.pdf]

SUPPLEMENTARY DATA

Supplementary Figure 1. Mitochondria morphology in mutant *TARDBP* and *C9ORF72* fibroblasts after 48 hours in galactose medium. Representative confocal images of mitochondrial network in primary fibroblasts from three healthy controls (C1, C2, C3), three ALS patients carrying mutation in *TARDBP* gene (p.A382T; T1, T2, T3) and three ALS//FTD patients with pathological expansion of the hexanucleotide repeat in *C9ORF72* (C9.2, C9.3, C9.4) transfected with pDsRed2Mito construct. Bar, 10μm.

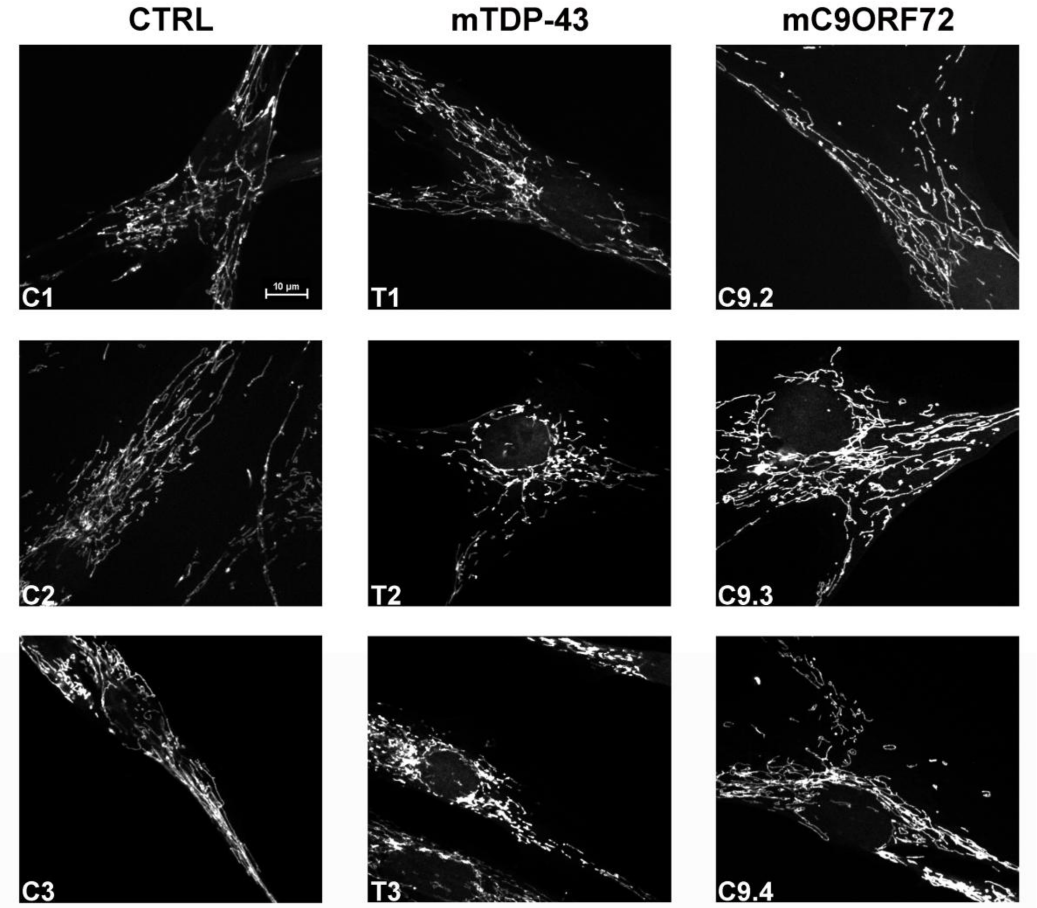

Supplementary Figure 2. Mitochondria functionality in mutant *TARDBP* and *C9ORF72* fibroblasts. Measurement of mitochondrial membrane potential by flow cytometry analysis of Mitotracker Red (MTR) positive fibroblasts (from 4 healthy controls, 3 *TARDBP* and 4 *C9ORF72* mutated patients) maintained in medium with galactose and without glucose. Median ± SEM, n=3 different *DIV*; One-way ANOVA with Dunnett's multiple comparison test; **p<0.01).

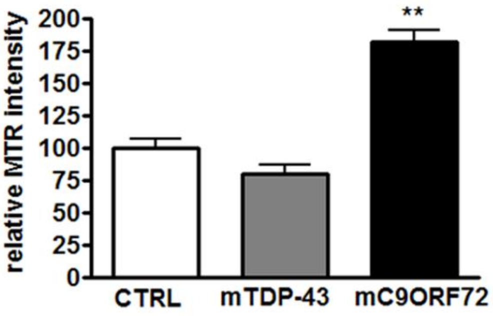

Supplementary Figure 3. Analysis of mitochondria membrane potential in mutant *TARDBP* and *C9ORF72* fibroblasts in glycolitic conditions. Measurement of mitochondrial (MMP) by flow cytometry analysis of TMRM-positive fibroblasts (4 healthy controls, 3 *TARDBP* and 4 *C9ORF72*), maintained in medium with 2 g/l glucose. Median ± SEM, n=3 different *DIV*; One-way ANOVA with Dunnett's multiple comparison test).

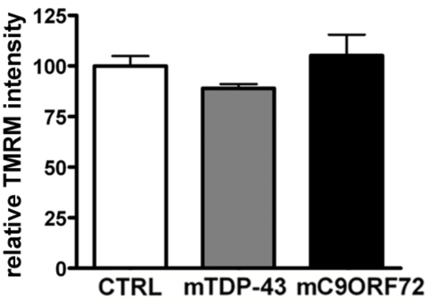

Supplementary Figure 4. Analysis of mitochondria mass in mutant *TARDBP* and *C9ORF72* fibroblasts in glycolitic conditions. Measurement of mitochondrial mass (MM) by flow cytometry analysis of Mitotracker green (MTG)-positive fibroblasts (4 healthy controls, 3 *TARDBP* and 4 *C9ORF72*), maintained in medium with 2 g/l glucose. Median ± SEM, n=3 different *DIV*; One-way ANOVA with Dunnett's multiple comparison test).

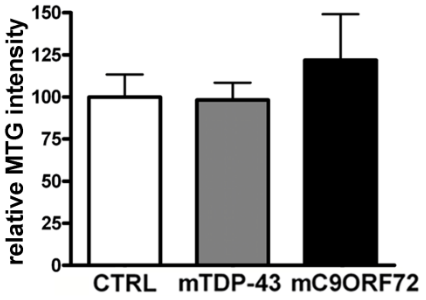

Supplementary Figure 5. TDP-43 sub-cellular localization in *C9ORF72* fibroblasts after 48 hours in galactose medium. Representative immunofluorescence images of TDP-43 (green) in primary fibroblasts from healthy controls (four) and ALS/FTD patients carrying mutation in *C9ORF72* gene (four), incubated with Mitotracker Red. Bar, 10 µm.

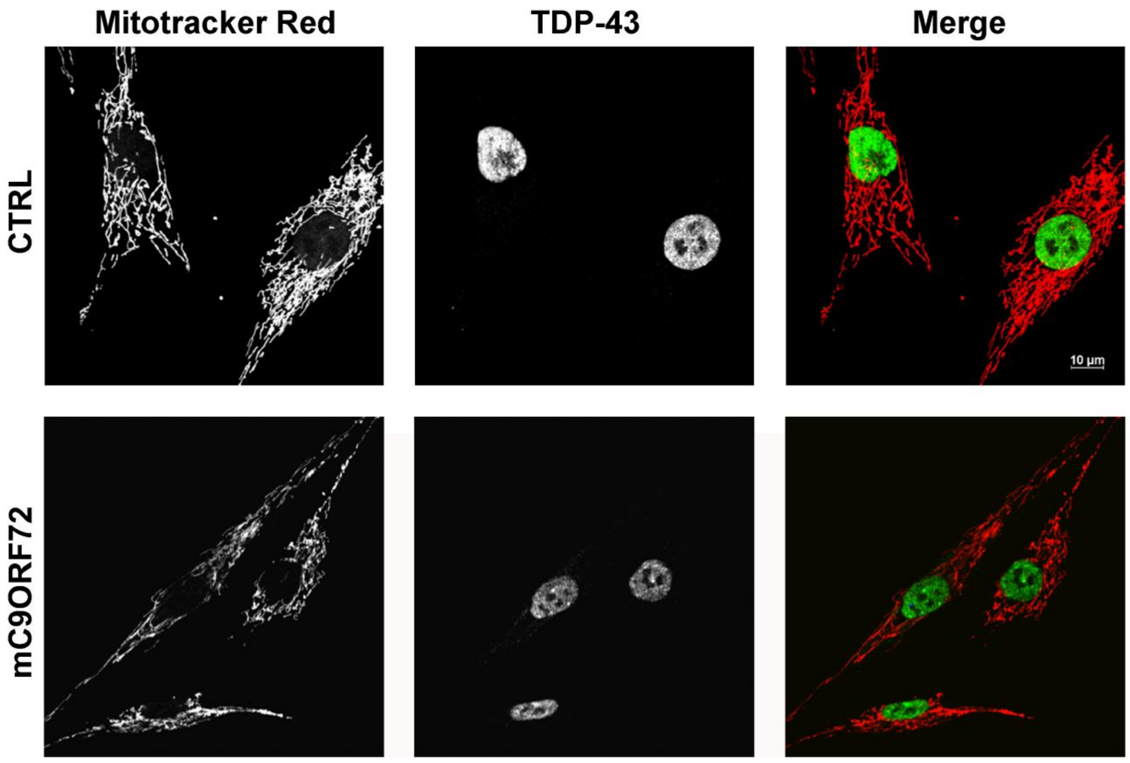

Supplementary Table 1. Clinical features of healthy controls, *TARDBP*- and *C9ORF72*-mutated patients

Patient ID	Sex	Age at biopsy	Gene	Mutation	Diagnosis	Family history	Age at onset	Site of onset	Cognitive impairment
**C1**	M	37	-	-	-	-	-	-	-
**C2**	M	46	-	-	-	-	-	-	-
**C3**	F	53	-	-	-	-	-	-	-
**C4**	M	62	-	-	-	-	-	-	-
**T1**	M	26	TARDBP	p.A382T	ALS	No	25	spinal	No
**T2**	M	56	TARDBP	p.A382T	ALS	No	54	spinal	No
**T3**	F	60	TARDBP	p.A382T	ALS	No	59	spinal	No
**C9-1**	F	57	C9ORF72	1500 units	ALS	Yes (sister)	55	spinal	No
**C9-2**	M	64	C9ORF72	1166 units	ALS	No	61	spinal	No
**C9-4**	M	69	C9ORF72	>4000 units	ALS	No	66	spinal	No
**C9-3**	F	69	C9ORF72	2300 units	FTD	Yes (son with MND)	68	-	Yes

Supplementary Table 2. Primer sequences and probes for Quantitative Real time PCR

MAP1LC3 FOR	CAGCATCCAACCAAAATCCC
MAP1LC3 REV	GTTGACATGGTCAGGTACAAG
P62 FOR	CCAGAGAGTTCCAGCACAGA
P62 REV	CCGACTCCATCTGTTCCTCA
MT-ND5 FOR	GCAGCCATTCAAGCAATCCTA
MT-ND5 REV	AGGCGAGGATGAAACCGATAT
MT-ND5 MGB FAM probe	ACAACCGTATCGGCG

Supplementary Table 3. Respiratory electron transport chain activity. Values are expressed as nmol/min/mg protein and normalised with citrate synthase activity.

	**Complex I**	**Complex II**	**Complex III**	**Complex IV**	**Succinate deidrogenase (SDH)**
**CTRL**	36,9±4,9	11,7±1,2	66,0±4,9	108,9±12,5	9,7±0,2
**mTDP-43.A382T**	38,0±7,56	14,1±0,3	60,7±3,2	133,0±7,9	11,7±0,6
**mC9ORF73**	34,9±6,1	13,8±0,6	76,2±10,0	116,2±12,4	12,9±1,0
